# Trelliswork and Craquelure

**DOI:** 10.1177/2041669517735125

**Published:** 2017-10-09

**Authors:** Jan Koenderink, Andrea van Doorn, Johan Wagemans

**Affiliations:** Department of Psychology, Justus Liebig University Giessen, Germany; Laboratory of Experimental Psychology, University of Leuven (KU Leuven), Belgium; Experimental Psychology, Helmholtz Institute, 8125 Utrecht University, the Netherlands; Department of Psychology, Justus Liebig University Giessen, Germany; Experimental Psychology, Helmholtz Institute, 8125 Utrecht University, the Netherlands; Laboratory of Experimental Psychology, University of Leuven (KU Leuven), Belgium

**Keywords:** grout color, outlining, craquelure, image fragmentation, cracks, partial occlusion, assimilation

## Abstract

Consider a mosaic image, the edges of the tesseræ being unrelated to pictorial content. Depending upon grout color, the picture is seen as uninterrupted “behind bars” or divided into tiles by “cracks” as in an ancient oil painting. The phenomenology is explored.

We serendipitously discovered a visually striking effect that we so far have not heard mentioned before—but we keep asking around.

In a number of experiments (Koenderink, Richards, & van Doorn, 2012a, 2012b) and demonstrations (e.g., ECVP 2012 *Illusoria Mente*; see Koenderink, 2012), we have used grids of lines that were apparently overlaid over images, but actually served to mask spatial discontinuities of piecewise local offsets. The grout was a medium gray and the line-thickness thin or moderate. Recently, it became desirable to vary the grout gray level, which is how we discovered the present effect.

Consider the images juxtaposed in Figure 1. The pictures have chromatic variations, whereas the grout is achromatic in both cases. This is one reason why the grout tends to be perceived as alien to the picture. Another reason for this is that the shape of the tile boundaries is not related to the pictorial content. This is no doubt that this is also an important factor. We explore such influences here. The effects work just as well for pictures that show abstract gray-level configurations (Figure 2).

**Figure 1.**
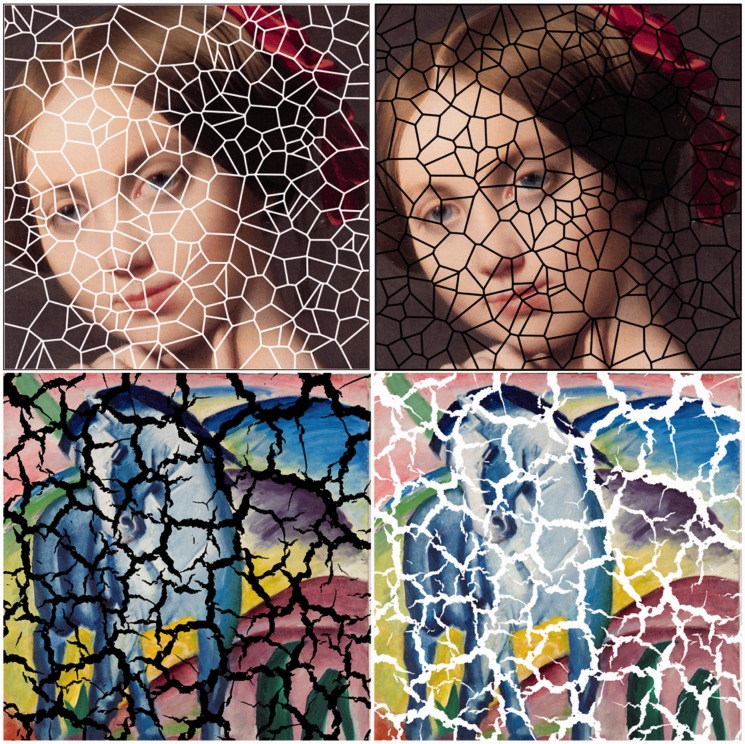
Top row: Detail from the painting “Vicomtesse Louise-Albertine d’Haussonville” by Ingres (1845, public domain). We superimposed random Poisson-Voronoi lattices in various gray tones. Note that in the image at left, where the lattice is white, the portrait is seen as unfragmented and extending *behind* the lattice, whereas the lattice appears to subtend a frontoparallel, open trelliswork in front of the painting. In contradistinction, in the image at right, where the lattice is black, the portrait is seen as fragmented by the mesh, whereas the lattice appears to partake in the picture plane in the form of a pattern of cracks. Bottom row: The “style” of the lattice is not too important. Here, we used a texture pattern of cracked leather. The painting is a detail of “Blue horse” by Franz Marc (1911, public domain). The black mesh at left appears as a craquelure fragmenting the picture plane. In contradistinction, the white lattice at right appears as a trelliswork in front of an integral image. This is perhaps more remarkable than the case of the top row, because the mesh-style explicitly depicts a craquelure texture.

**Figure 2.**
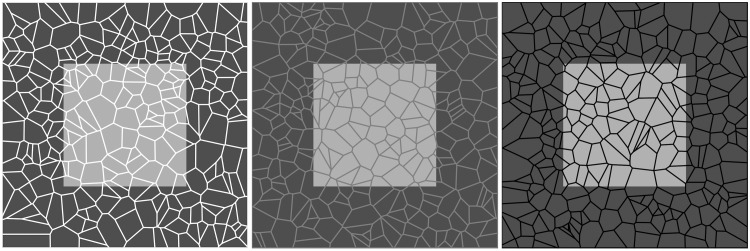
The effect seen in Figure 1 top row appears also for the simplest artificial configurations as here a light square on a dark ground. The square at left appears as an integral object behind a white lattice, whereas the square at right looks fragmented, being tessellated by the grid. In the case at left the square and the lattice appear in different depth planes. Curiously, the square looks lighter because of the assimilation (von Bezold, 1874; Musatti, 1953) induced by the white grid. Apparently assimilation works across depth planes, which is a notable finding by itself (Agostini & Galmonte, 2000). In the figure at center, unlike those at left or right, the mesh appears dark on some parts and light on the other. The square appears to lie behind a trellis, the latter appearing in front of both the square and the dark background. This is the condition used in most of our demos and experiments (Koenderink et al., 2012a, 2012b).

The following generic forms of visual awareness could conceivably arise:

– the picture could disintegrate into mutually unconnected, although related, tiles. Here, we concentrate on the case of *craquelure* as it often occurs in paintings. These are *cracks* that are unrelated to the pictorial content;
– the picture could appear as a mosaic, perhaps like a stained glass window. The pieces would indeed be fragments, but “glued together” by the *grout*. The seams may or may not relate to the pictorial content;
– the picture could appear as unscathed, running on *behind a trelliswork* so to speak, the lines not appearing as grout, but as an open trellis *in front of* the picture. There would be an obvious depth relation where the trelliswork and the picture would appear unrelated and mutually disconnected.

In our previous work (Koenderink et al., 2012a, 2012b), we invariably encountered the latter case, perhaps surprisingly, even when the visible tiles were mutually offset. This effect shows that the tendency to perceive the pictorial content unbroken behind an open trellis is very strong and not easily counteracted.

The second and third cases are realized for *the same picture*, and* the same mesh,* merely depending upon the gray level of the grout. With the black grout, we perceive a “cracked” picture a bit like an old oil painting showing craquelure of the varnish. With the white grout, the picture apparently has an integral surface that runs on behind a trelliswork in front of it. At first blush, the difference is striking. However, with some effort, one is sometimes able to switch between the two appearances. We found that for some observers, the effect is bistable. Some observers are able to voluntarily “flip.”

In Figure 3, the fragments are fitted to the figural content. The effect becomes considerably weaker and depends on the way one views the image. Apparently the fit of the cracks to the figural edges is important. However, this is not obviously related to the influence of the color of the cracks.

**Figure 3.**
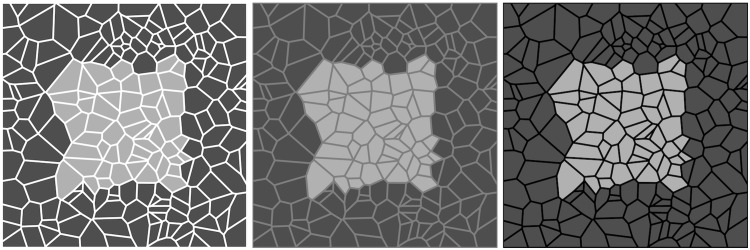
Unlike in Figure 2, here the mesh fits the figural content. If one looks *globally* the effect is still present, though weakened. If one looks *focally*, attending to the fragments and their boundaries, it is absent. Like in Figure 2, the effect of assimilation is evident, no matter how one attends to the picture.

The finding has various potential applications in visual arts and design, and in art history. Cracks due to technical constraints (mosaics, Dunbabin, 1999; stained glass windows, Sowers, 1965) or artistic intention (*cloisonnism* in painting, Dujardin, 1888) as well as cracks due to aging (*craquelure* in aged oil paintings, Bucklow, 1999) and Japanese *kintsugi* (the repair of broken pottery with gold-colored cement, Jobson, 2014) come to mind.

Various speculations as to the origin of the effect readily come to mind, but for this short communication we leave it at that.
